# Public Health Considerations Associated with the Location and Operation of Off-Leash Dog Parks

**DOI:** 10.1007/s10900-017-0428-2

**Published:** 2017-10-12

**Authors:** Tissa Rahim, Pablo Romero Barrios, Geoffrey McKee, Melissa McLaws, Tom Kosatsky

**Affiliations:** 1 British Columbia Centre for Disease Control, 655 W 12th Avenue, Vancouver, BC V5Z 4R4 Canada; 2 School of Public and Population Health, University of British Columbia, Vancouver, BC Canada; 3 Environmental Health Services, Vancouver, Canada; 4 Canadian Food Inspection Agency, Vancouver, Canada; 5 Public Health and Preventive Medicine, Vancouver, Canada; 6 National Collaborating Centre for Environmental Health, Vancouver, Canada

**Keywords:** Public health, Dog parks, Off-leash, Urban planning, Built environment

## Abstract

Off-leash dog parks may enhance human health, but may also lead to health risk through infection or canine aggression. Published evidence was reviewed to examine positive and negative public health impacts of off-leash dog parks, as well as strategies for enhancing benefits and mitigating risks. Evidence suggests that off-leash dog parks can benefit physical and social health, as well as community connectedness. While studies have documented shedding of zoonotic agents in dog parks, the risk of transmission to humans is relatively unknown. Evidence on the risk of dog bites in off-leash dog parks is also limited. Case-examples from North American off-leash dog parks highlight the importance of park location/design, public adherence to safe and hygienic practices, and effective regulatory strategies for mitigating potential risks and maximizing the benefits of off-leash dog parks.

## Introduction

Off-leash dog parks are public spaces where dogs can exercise off-leash in designated areas under the supervision of their owners [1]. The prevalence of dog ownership (32% of Canadian households) [2], along with increased urbanization and density, have increased the demand for off-leash dog parks; however, off-leash dog parks have triggered controversy since their introduction to North America in 1979 [3]. Proponents often value access to areas where their dogs can exercise and socialize, while opponents cite concerns about public safety and nuisance [4–7]. Off-leash dog parks are of particular interest in health promotion because they may enhance physical activity and social networking for some individuals, while deterring park use for others [1].

In Canada, jurisdiction over dog park development and maintenance generally falls to municipal governments [8]. Off-leash dog parks can be established either through allotment of space in existing parks, or the creation of new parks; the former is the more common approach [9]. In these parks, by-law officers enforce rules on dog access and activities established by municipal parks boards and animal control agencies [8].

As part of creating new off-leash dog parks, it is important to identify and prevent potential health risks, and to maximize the health benefits to both dogs and owners. Public health departments can play an important advisory role in land use planning and related activities, and may be asked to provide comments on the potential impacts of off-leash dog parks on public health. This review aims to summarize published evidence examining positive and negative public health impacts of off-leash dog parks, and to lay out strategies for enhancing benefits and minimizing harms. For the purposes of this paper, canine health and ecological/environmental factors were not addressed.

## Methods

A comprehensive review of the scientific literature was conducted using electronic databases, including CAB Direct, Google Scholar, Medline, PubMed, and Web of Science. Non-academic resources were also consulted, including government, public health department and newspaper websites. Bibliographies of retrieved articles were reviewed to locate additional material relevant to the search criteria. Based on the requirements of the database searched, both controlled terms and free text were used. The general search included seven key terms combined using boolean operators (i.e. “and” and “or”): (1) dog park; (2) public health; (3) policy; (4) risk; (5) off-leash; (6) canine; (7) health. Search results were restricted to English-only articles; no date restrictions were applied.

## Results

### Literature Search

Our literature search yielded 203 articles of interest, of which 176 were peer reviewed journal articles, and 27 came from the grey literature. Twenty-nine articles were reviews of canine and human health/public health/policy. Overall, it was difficult to identify studies with isolated assessments of dog ownership, dog walking and dog park utilization, as many studies examined these topics in combination. We identified 53 primary research articles studying the effects of dog ownership and/or dog walking on physical and/or social health. Articles included studies of off-leash dog parks (14), dog fouling (5), zoonoses (16), dog bites and dog aggression (2), and dog ownership/dog walking (16).

### Health Benefits

#### Physical Benefits

Inactivity is an important risk factor for many human chronic diseases, including heart disease, hypertension, obesity and diabetes [10, 11]. 48% of Canadians aged 12 and older are considered to be inactive with respect to Canadian physical activity guidelines [12]. Dog walking represents an opportunity to maintain a moderately active lifestyle and meet recommended physical activity guidelines [13–17]. It is also associated with lower risk of hypertension, depression, and death following myocardial infarction [15, 18–20]. Several studies have demonstrated that frequent dog-walkers are more likely to achieve recommended levels of physical activity compared to infrequent dog-walkers and non-owners [13, 21–29].

Six studies examined levels of physical activity in off-leash dog parks and reported mixed results. Dog parks were reported as one of the most commonly used types of parks in Southern California, although leash requirements were not mentioned [30]. An observational study of six dog parks with designated off-leash hours in Victoria, British Columbia (BC), found that dog owners maintained their walking practices more often than non-dog owners even during inclement weather [26]. Similarly, a cross-sectional telephone survey in Calgary, Alberta, reported that the frequency of dog walking was higher among dog owners who resided within 1.6 km of an off-leash area, compared to other dog owners [17]. Proximity to off-leash dog parks was also correlated with increased frequency of use in an observational study in Texas and Florida [31]. In another observational study of off-leash dog parks in the United States (US), dog walking was more common among more frequent park visitors; however, duration of stay in the park was shorter [32].

In contrast, a greater proportion of dog-walkers were observed to be stationary (i.e. dog owners who stood or sat while their dogs ran free) in two parks in Calgary, Alberta, when the areas were designated as off-leash [33]. These findings have been replicated in other Canadian and US studies [26, 34]. It has been hypothesized that the decreased mobility of dog-owners in off-leash parks may be due to owners socializing rather than walking with their dogs [15].

#### Social Benefits

Dog ownership and the use of dog parks have also been studied in the broader context of community and social health [35]. Off-leash parks introduced in sparsely-used areas have been associated with a subsequent reduction in locally reported criminal activity [36, 37]. This was also observed in parks that are designated off-leash only during off-peak hours (i.e. evenings/nighttime and during the winter season) [36, 37].

Additionally, off-leash dog parks may improve social connectedness and overall community satisfaction by “catalyzing” social interactions [38]. The health risks attributed to the lack of social relationships are comparable to cigarette smoking, elevated blood pressure and lack of physical activity [35, 39, 40]. Enhanced social capital (i.e. positive networks of relationships in the community), community satisfaction and higher neighborhood safety appear to have considerable, indirect effects on individual human health, and may be facilitated by dog ownership and use of dog parks.

In a random telephone survey in Calgary, Alberta, older adults (over 50 years of age) who frequently walk their dog reported more positive feelings about their neighborhoods and an enhanced sense of community [21]. Similarly, in observational studies in Texas, Florida, and Georgia, community members reported off-leash dog parks increased socialization with neighbours and created a heightened sense of community [6, 31]. Wood and colleagues (2007) described a “ripple effect” of dog ownership and associated park use on neighborhood interactions and sense of community that could extend beyond dog owners to the broader community [41]. Elderly individuals or those with physical disabilities, for whom off-leash dog parks promote the formation of new social bonds as a side-effect of canine exercise, may particularly benefit [39]. The increase in community social connectedness could also have an indirect effect on responsible dog ownership, as owners share knowledge from their own experiences regarding pet hygiene and safety; however, these connections could also result in tension and exclusion of owners with poorly behaved dogs [42].

### Health Risks

Common concerns regarding off-leash dog parks include dog fouling, zoonotic infections, bites, noxious smells, noise, unruliness, and fear that dogs may act aggressively [37, 43].

#### Dog Fouling

Dog fouling, or failure to remove dog waste, is an oft-raised nuisance issue, which can also result in adverse health consequences [44]. There is concern that introducing off-leash areas could lead to increased dog-fouling due to greater density of dogs in designated park areas and reduced owner vigilance [4]. Not only is the presence of dog feces aesthetically unappealing, undisposed feces can lead to slips, falls, and subsequent injuries, as well as the transmission of zoonotic agents [43, 45]. While this may be a common complaint, there is limited evidence for greater dog fouling in off-leash dog parks compared to those with on-leash requirements. Rock et al. found implementation of off-leash policies resulted in conflicting results in two parks in Calgary, Canada [33]. Increased compliance with rules requiring proper disposal of dog waste was observed in one park, but not another, when compared to the same park prior to implementation [33].

#### Zoonoses

Dogs can carry a variety of human pathogens, including *Escherichia coli, Campylobacter jejuni, Salmonella *spp., and *Giardia lamdia* (Table 1) [45–60]. These may be transmitted to humans, either directly through contact with infected dogs or indirectly via exposure to feces, urine and/or contaminated water or environments [60]; however, risk to human health depends on various factors, such as pathogenicity of the organism, concentration in feces, and route of exposure [50].

Given that off-leash designations may enhance dog–dog interactions, Westgarth et al. suggested there may be elevated risk of transmission of zoonotic agents [61]. While studies have examined the shedding of zoonotic agents in Canadian dog parks, none has explored the transmission risk from dogs to humans; however, dog ownership has been investigated as a risk for zoonotic transmission. In a US study, dog ownership was associated with increased *Toxocara* seropositivity (odds ratio: 1.2, 95% CI 1.1–1.4) [62]. Another study from the US reported an increased likelihood of *Cryptosporidium* infection in HIV-positive individuals who owned dogs as compared to those who did not (odds ratio: 2.19, 95% CI 0.9–5.3) [63]. Nonetheless, these studies may not be representative of the risk of *Toxocara* or *Cryptosporidium* infections in the general population associated with contact with off-leash parks.

A study in Calgary city parks found a parasite prevalence among canine fecal samples of 50%; 25% contained *Giardia* spp., 15% *Cryptosporidium* spp., 17% *Cystoisospora* spp., and 4% helminths [64]. The prevalence of parasite infection was positively associated with dogs visiting multiple parks, as well as off-leash activity [64]. Another study of *Giardia* spp. among urban parks in Calgary demonstrated a significant, positive association between the presence of *Giardia* spp. among dogs and off-leash area use, as well as dog swimming frequency [49]. A study of off-leash dog parks in South-Western Ontario reported a prevalence of *Salmonella, Giardia* and *Campylobacter* spp. in fecal samples of 1, 6, and 43%, respectively [50]. In particular, younger and older dogs appeared to be at highest risk of shedding *Campylobacter* spp. [50]. Fecal samples collected from dog walking areas (including off-leash dog parks) in Saskatoon, Saskatchewan, demonstrated a variety of potential pathogens, including roundworm species (2%), hookworm species (0.4%), whipworm species (0.7%), and *Strongyloides* spp. (0.6%), as well as *Giardia* spp., *Cystoisospora* spp., and *Alaria* spp. in 0.4% of samples [65].

**Table 1:** Selection of canine zoonoses [66]

Brucellosis
Campylobacteriosis
Cryptosporidiosis
Dermatophytosis (ringworm)
*Escherichia coli*
Echinococcosis
Ehrlichiosis
Giardiasis
Leptospirosis
Pasteurellosis
Rabies
Salmonellosis
Sarcoptic mange
*Staphylococcus * spp.
Strongyloidosis
Toxocariasis

#### Canine Aggression

Canine aggression and risk of bites are often cited by opponents of off-leash dog parks, particularly given the limited control over unrestrained dogs [4]. In addition to immediate injury, bites may represent a considerable health concern due to the possibility of secondary infection and/or mental health sequelae [67, 68]. However, the frequency of dog bites to humans in parks, including off-leash dog parks, has not been studied. Reviews of dog bite injuries from the US, Canada, and Australia have reported that a majority of dog bites occur in the home [68, 69]. For children under 15, emergency department surveillance from Australia found that 66% of dog bite injuries occurred in the patients’ own homes or a home they were visiting, and only 19% occurred in public places [69]. Children may be more susceptible, as they are approximately 3–5 times more likely to experience dog bites than adults [67, 68]. These injuries may also be more severe, as children are more likely to experience bites involving the head, neck, and face [67, 68].

## Discussion

Although the literature supports the health benefits of dog ownership and dog walking, there is insufficient evidence to fully characterize the specific risks and benefits of off-leash dog parks. There are many studies investigating the effect of dog ownership on human health, but limited research into off-leash dog parks, their location/area type, and influence on public health. Many studies have also been limited to Caucasian dog owners of middle to higher socioeconomic statuses, indicating potential ethnographic/demographic biases. Finally, the preponderance of studies reliant on qualitative data and self-reporting makes generalizability and comparisons challenging. Nonetheless, the existing literature may be supplemented with examples of successful implementation in order to inform a discussion of measures that can be taken to maximize potential benefits and minimize harms of off-leash dog parks.

### Strategies to Maximize Benefits

Various park characteristics have been associated with increased physical activity among dog owners. Park features, such as a linear or walkthrough design, may deter sedentary behaviour by encouraging dog-owners to walk alongside their dogs [26]. Living near a designated off-leash area, and provision of dog litterbags and dog-related signage may also enhance dog-walking frequency [17, 70–72]. Proper park maintenance and enhanced safety (including neighborhood traffic volume and speed restrictions, park lighting, and reduced crime levels) appear to influence the likelihood of use by dog walkers [26, 73]. Durable, low-maintenance seating that faces the off-leash area can facilitate the social benefits of off-leash dog parks by balancing owner conversation with dog supervision [42].

Alternatively, some on-leash parks also allow dogs to be walked off-leash during off-peak hours or less busy months in order to avoid safety concerns [36]. This approach also been associated with a reduction in criminal activity, as these parks continue to be frequented at off-peak hours [36, 37]. Many parks also fence off their designated off-leash areas and limit access to other parts of the park to on-leash dogs, allowing park goers to avoid unrestrained dogs if preferred [9].

### Strategies to Minimize Harm

Choice of location is key to ensuring safety, community satisfaction, and effective operation of the park. In order to mitigate safety concerns, particularly for vulnerable individuals such as children, off-leash dog parks should not be located directly adjacent to playgrounds or schools, nor interfere with established park uses [37].

The design of dog parks can also limit the degree of potential risk. Secure fencing (i.e. a gated enclosure at least four feet high) may protect park users, including children and cyclists, from aggressive dogs in addition to setting a clear boundary [74]. Provision of dog waste bags, access to waste receptacles that are routinely emptied, and signage reminding owners to pick up after their dogs, can reduce dog fouling [74]. The availability of hand-sanitizing stations for dog park attendees can also reduce risk of disease transmission [74]. Clearly visible rules, as well as following the example of fellow dog walkers, may result in improved compliance [33].

Quick removal of dog feces may significantly reduce the likelihood that parasites incorporate into soil, greatly reducing the likelihood of transmission [3]. Given that access to and contamination of water sources, including lakes and ditches, may increase the likelihood of disease transmission from dogs to humans [75], off-leash dog parks should be located away from sources of standing water and run-off [76]. Public messaging may advise owners that, if a dog is ill and/or known to be infected by a zoonotic pathogen, they should avoid walking them in busy park areas and bodies of water until treatment for the infection is completed [60].

Education and awareness initiatives may also mitigate behaviours that increase risk of harm to dog owners and other park attendees. Encouraging responsible dog ownership (i.e. maintaining continuous vigilance over their pet during a park visit) and hygienic practices can help avoid risk of aggression/injuries and transmission of zoonotic pathogens [74, 76]. Signs reminding attendees of park rules should be clear and visible, placed at park entrances, and include simple messages. These may also include maps of the designated off-leash zone, and contact information for reporting damage or making a complaint [3]. Signs should emphasize the importance of hand washing and proper disposal of dog waste [77]. Additional instructions may advise constant supervision and verbal control of the dog at all times, as well as the need for dogs to wear a valid license and be up-to-date on their vaccinations. It may also be beneficial to include messaging for owners to muzzle aggressive dogs and ensure they retain a leash in hand at all times while the dog is running free in cases where leashing may be required (i.e. if the dog is exhibiting aggressive behaviour) [77]. Finally, signs should remind owners they are responsible for any damage or injury inflicted by their dog [77].

Patrolling officers should be available to enforce policies that prevent rule violations, such as dog fouling and allowing aggressive dogs to remain unrestrained [74]. Rules and regulations should be publicized, whether through signage or publication on a park-related website [76]. Websites may also provide information on zoonoses and encourage regular veterinary check-ups [60]. Veterinary assessment may identify risk factors and symptoms of zoonoses in dogs, with regular visits ensuring proper vaccination, regular deworming, and provision of health and hygiene messaging [60]. As an example, the American Veterinary Medical Association published a pamphlet entitled “Internal Parasites in Cats and Dogs” that contains information on the most common parasites, detection methods and tips for prevention [78].

In addition to education of dog owners, other park attendees, such as children, may benefit from initiatives that enhance their ability to interact with dogs in a way that is less likely to result in aggression or injuries [60, 79, 80]. For example, educational programs in schools and children’s museum settings on basic safety rules, as well as interactive computer animations and picture books, have been designed to educate children and families about properly interacting with dogs to minimize risk of injury [80].

### Public Consultation and Evaluation

In order to address public concerns about the potential risks of proposed off-leash dog parks, decision-makers should be proactive and ensure broad community consultation. Clear descriptions of proposed plans for off-leash areas should be published in order to facilitate public feedback. Various stakeholders, including dog owners, non-dog owners, adjoining property owners, civic leagues, and animal health agencies should be consulted prior to initiating off-leash dog park development [81].

Ongoing, bidirectional communication between municipal governments and stakeholders may also alleviate concerns, prevent conflicts, and ensure continued community satisfaction/safety once an off-leash area has been opened. Mechanisms allowing park users and nearby residents to communicate park-related concerns with the relevant officials may inform ongoing park evaluation and improvements in response to perceived risks. This could be achieved via online polls, email lists, or scheduled meetings, as well as other technologies, including texting or phone applications [76, 77].

### Case Studies

See Table 2.

**Table 2:** Overview of five municipal off-leash dog park initiatives in Canada and the US

Location	Implementation	Outline of recommendations
Kelowna, British Columbia	Incorporated two off-leash dog parks on a 2-year trial basis [82]	Community input
		Continuous monitoring and evaluation
		Limit dog access in areas where human recreational activities occur
		Access to hand-washing facilities
Surrey, British Columbia	Created a “Dog Off Leash Area Strategy” to guide development of off-leash dog parks over 2011–2021 [76]	Summary of construction practices and materials used
		Quick waste disposal
		Requiring documentation of veterinary check-ups
		Easy-to-read rule enforcements
Calgary, Alberta	Created an updated off-leash management plan following extensive public engagement through surveys and open houses [83]	Evaluation of existing off-leash areas
		Volunteer program to assist with educating dog owners about dog-related bylaws and off-leash etiquette
Edmonton, Alberta	Created a “Dogs in Open Spaces Strategy”, a 10-year strategy outline [84]	Planning recommendations: location choice based on population density and dog ownership numbers
		Design recommendations: setting of boundaries/fencing and walking circuits that encourage owners to keep moving with their dogs
		Management recommendations: educating park users through signage, enforcement of rules, and regular monitoring and evaluation
Seattle, Washington	Identified 70 possible off-leash sites; a 15 month pilot program was implemented at eight of those sites [37]	Avoid locations near children’s play areas
		Avoid spill-over into non-dog areas

## Conclusion

Although the associations between dog ownership or park use and health behaviours have been widely studied, off-leash parks have received limited attention. Nonetheless, available evidence suggests that off-leash dog parks can benefit physical and social health, as well as community connectedness. By considering the impact of park location/design, promoting public adherence to safe and hygienic practices, and employing effective regulatory strategies, municipalities can mitigate potential risks and maximize the benefits of off-leash dog parks on community health and wellbeing.
